# 

**Published:** 1872-10

**Authors:** 


### Contents of October Number.

### ORIGINAL DEPARTMENT.

ART. I.—Forensic Medicine. By Charles B. Knowlton,.............. .. 81
ART. II.—A Case of Protrusion of the Spleen through an opening in the Abdomen. By W. H. Williams, M. D.,........................ 86
ART. III.—Surgical Cases treated in the Buffalo General Hospital.. By C. C. F. Gay, M. D.,............ ................................. 88
ART. IV.-Obstetrics in the Sandwich Islands. By Chas. H. Wetmore, M.D. 90

### TRANSLATIONS.

On the Recent Advances in the Theory of Vision. By H. Helmholtz. Translated by F. W. Abbott, M. D.,.............................. 94

### MISCELLANEOUS.

Abstract of a Clinical Lecture on Death from Chloroform. By Eric Erichsen,..................................................101
A Successful Case of Immediate Transfusion.........................103
Cure of Strangulated Hernia by Aspiration..........................105
Grafting with Rabbit Skin..........................................106
Apparent Death,....................................................106
Items and Selections. By E. N. Brush...............................107
Death from Stings of Bees..........................................109
Death from Ether.................................................. 110
Poisoned Wounds from the Bite of the Snake and Tarantula, and the Sting of the Scorpion and Centipede.................................111

### EDITORIAL DEPARTMENT.

Epidemic Catarrh among Horses......................................113
Recent Advances in the Theory of Vision............................114

BOOKS REVIEWED:

Diseases and Displacements of the the Uterus. By Edwin N. Chapman, M. D.........................................114
The Physiology of Man, the Nervous System. By Austin Flint, Jr., M. D...........................................115
On Food. By H. Letheby, Ph. D. etc.,.......................115
Thermic Fever, or Sunstroke. By H. C. Wood, Jr., M. D.....116
The Ten Laws of Health. By J. R Black, M. D.,.............117
Hallucinations of Childhood. By H. M. Knight, M. D.,.......117
Annual Report of the Connecticut School for Imbeciles.......118
Proceeding of the Royal Society............................118
Report on Practical Medicine. By T. D. Washburn, M. D.....118
Transactions of the Pa. State Medical Society..............119

Literary. Introductory Lecture.....................................119
Books and Pamphlets Received.......................................120

THE BUFFALO

